# Stepping Out of the Shade: Control of Neuronal Activity by the Scaffold Protein Kidins220/ARMS

**DOI:** 10.3389/fncel.2016.00068

**Published:** 2016-03-14

**Authors:** Joachim Scholz-Starke, Fabrizia Cesca

**Affiliations:** ^1^Institute of Biophysics, Consiglio Nazionale delle RicercheGenova, Italy; ^2^Center for Synaptic Neuroscience, Istituto Italiano di TecnologiaGenova, Italy

**Keywords:** Kidins220/ARMS, BDNF, sodium channels, glutamate receptors, synaptic plasticity, neuronal excitability, neurodegeneration

## Abstract

The correct functioning of the nervous system depends on the exquisitely fine control of neuronal excitability and synaptic plasticity, which relies on an intricate network of protein-protein interactions and signaling that shapes neuronal homeostasis during development and in adulthood. In this complex scenario, Kinase D interacting substrate of 220 kDa/ankyrin repeat-rich membrane spanning (Kidins220/ARMS) acts as a multi-functional scaffold protein with preferential expression in the nervous system. Engaged in a plethora of interactions with membrane receptors, cytosolic signaling components and cytoskeletal proteins, Kidins220/ARMS is implicated in numerous cellular functions including neuronal survival, neurite outgrowth and maturation and neuronal activity, often in the context of neurotrophin (NT) signaling pathways. Recent studies have highlighted a number of cell- and context-specific roles for this protein in the control of synaptic transmission and neuronal excitability, which are at present far from being completely understood. In addition, some evidence has began to emerge, linking alterations of Kidins220 expression to the onset of various neurodegenerative diseases and neuropsychiatric disorders. In this review, we present a concise summary of our fragmentary knowledge of Kidins220/ARMS biological functions, focusing on the mechanism(s) by which it controls various aspects of neuronal activity. We have tried, where possible, to discuss the available evidence in the wider context of NT-mediated regulation, and to outline emerging roles of Kidins220/ARMS in human pathologies.

## Introduction

To maintain the proper function of the nervous system, neuronal excitability and synaptic plasticity are continuously modulated in response to endogenous activity and external stimuli. A constantly increasing number of signaling and adaptor proteins form a network that contributes to maintain the balance between homeostatic compensation and experience-induced modifications during development and in adulthood. The picture however is still far from being complete, and new players are continuously added to this already complex scenario. Amongst such “new entries”, the Kinase D interacting substrate of 220 kDa/ankyrin-repeat-rich membrane spanning (Kidins220/ARMS, henceforth referred to as Kidins220) is a membrane protein preferentially expressed in the nervous system, where it modulates several crucial aspects of neuronal physiology such as cell survival, differentiation into axons and dendrites, and synaptic plasticity (Neubrand et al., 2012). Kidins220 is a large four-pass membrane protein that acts as a scaffolding protein and signaling platform at the plasma membrane. Its long amino (N)- and carboxy (C)-terminal tails are exposed to the cytoplasm and contain a number of protein-protein interaction modules that mediate most of the known Kidins220 functions. The N-terminal region is largely occupied by 11 contiguous ankyrin repeats that mediate the interaction with the Rho-guanine exchange factor (Rho-GEF) Trio and the rearrangements of the actin cytoskeleton triggered by nerve growth factor (NGF) signaling (Neubrand et al., 2010). The C-terminal tail contains several residues subjected to post-translational modifications, including phosphorylation, ubiquitination and calpain cleavage, and regions modulating the binding to a number of molecular interactors amongst which adaptor proteins of the mitogen-activated protein kinase (MAPK) pathway (Proline-rich domain; Arévalo et al., 2006), the molecular motor kinesin-1 [kinesin-interacting motif (KIM)] (Bracale et al., 2007), and the neurotrophin (NT) receptor p75^NTR^ [PSD-95/Disc large/Zonula occludens-1 (PDZ)-binding motif] (Kong et al., 2001). Other interactions, e.g., with α-amino-3-hydroxy-5-methyl-4-isoxazolepropionic acid (AMPA) receptor subunits and tropomyosin-related kinase (Trk)-type NT receptors, appear to be mediated by its trans-membrane domains (Arévalo et al., 2004, 2010). Kidins220 does not possess a catalytic function itself, but the interaction with target proteins is modulated by phosphorylation at multiple sites. Kidins220 is ubiquitously expressed in the central nervous system (CNS) and peripheral nervous system (PNS), being present in excitatory and inhibitory neurons, as well as in glial cells. At the subcellular level, Kidins220 shows no preferential localization or targeting, as it is present all over the cell body, dendrites and axons. Over the years, the majority of studies have focused on the role played by Kidins220 in the modulation of the intracellular signaling cascades initiated by NTs, in particular by brain-derived neurotrophic factor (BDNF) binding to its cognate receptor, TrkB. An increasing amount of data indicates that this protein plays different roles in the control of neuronal activity, which are cell- and context-specific, and that dysregulation of its function can lead to pathological consequences. In this review, we summarize the available data on the subject. This effort will hopefully help putting into context the various functions of Kidins220 in the modulation of neuronal communication and excitability, also and particularly with regard to NT-mediated regulation, which is at present far better understood.

## Kidins220 and the Regulation of Neuronal Activity

In addition to their classical functions in neuronal survival and differentiation, NTs—and in particular BDNF—have also been recognized as potent modulators of neuronal activity, working at multiple levels ranging from synapse formation and morphology to the dynamic modification of synaptic efficacy and membrane excitability (Poo, 2001; Blum and Konnerth, 2005; Gottmann et al., 2009; Edelmann et al., 2014). As a distinct down-stream target of activated NT receptors, it may not surprise that Kidins220 has been implicated in the molecular mechanisms controlling neuronal activity. In only few cases, however, the role of Kidins220 has been directly related to BDNF signaling events, while it mostly appears to function independently of BDNF, or at least, this relation has not been investigated.

These studies have relied on acute modifications of Kidins220 expression level by overexpression or gene silencing and the use of two independent Kidins220 knockout mouse lines. Homozygous knockout mice described by Wu et al. (2009) die very early during embryonal development, precluding further studies. Despite grossly normal brain development, heterozygous *ARMS*^+/−^ mice having 60–70% of normal Kidins220 protein levels were used as a model for reduced Kidins220 expression (Wu et al., 2009). Studies on the effects of constitutive *Kidins220* ablation have been possible with the generation of a second *Kidins220* knockout mouse line by Cesca et al. (2011, 2012), in which embryos survived until late stages of gestation. These embryos showed, among other phenotypes, a high degree of neuronal cell death in the CNS and impairments in the neuronal responses towards neurotrophic stimuli (Cesca et al., 2011, 2012).

### Roles of Kidins220 in Synaptic Transmission and Plasticity

**Figure 1:**
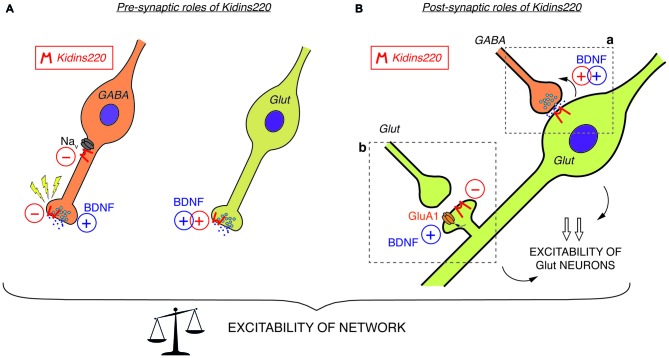
**(A)** *Pre-synaptic roles of Kidins220*. In GABAergic neurons (left), Kidins220 exerts negative effects on Na_v_ channel activity and on the recovery of neurotransmitter release upon stimulation. In glutamatergic neurons (right), Kidins220 favors the brain-derived neurotrophic factor (BDNF)-dependent stimulation of glutamate release. **(B)** *Post-synaptic roles of Kidins220 (in glutamatergic neurons)*. **(a)** At synaptic contacts between pre-synaptic inhibitory neurons and post-synaptic excitatory neurons, the presence of Kidins220 in the post-synaptic compartment favors the BDNF-dependent potentiation of pre-synaptic strength, possibly by modulating TrkB-dependent retrograde signaling events. **(b)** At synaptic contacts between pre-synaptic excitatory neurons and post-synaptic excitatory neurons, the presence of Kidins220 at dendritic spines negatively regulates the incorporation of the amino-3-hydroxy-5-methyl-4-isoxazolepropionic acid (AMPA) receptor subunit GluA1 into the plasma membrane, apparently in the opposite manner compared to BDNF, which is known to potentiate excitatory synapses by increasing the number of GluA1 at the membrane. Altogether, these effects are expected to impact on the excitation/inhibition balance, and consequently on network excitability. For both panels, please refer to the main text for a detailed discussion of possible mechanisms involved and for references to the published literature. Kidins220 action is represented as a red *minus* “−” when Kidins220 inhibits the process, and as a red *plus* “+” when Kidins220 favors the process. On the basis of the published literature, the role played by BDNF in the same phenomena is also represented as a blue *plus* symbol.

Among the studies conducted so far on this topic, there are only two examples directly investigating the relationship to TrkB/BDNF signaling. Both reports investigated well-known BDNF effects acting on the efficacy of synaptic vesicle release. Firstly, Sutachan et al. (2010) transiently altered Kidins220 levels in rat hippocampal pyramidal neurons (by over-expression and knock-down approaches) and found corresponding changes in the strength of gamma-aminobutyric acid (GABA)ergic inputs arriving at these neurons (Sutachan et al., 2010). Notably, the enhancement of GABAergic transmission in neurons chronically exposed to BDNF, which is known to boost GABA synthesis and release in inhibitory synapses (Bolton et al., 2000; Baldelli et al., 2002; Ohba et al., 2005), was completely abolished by *Kidins220* knock-down. In the second case, the stimulation of excitatory post-synaptic currents (EPSCs) elicited by acutely applied BDNF (Levine et al., 1995; Lessmann and Heumann, 1998) was impaired in cultured *Kidins220^−/−^* hippocampal neurons, in line with the decreased neuronal sensitivity towards neurotrophic stimuli in this mouse strain (Cesca et al., 2012). While both pre- and post-synaptic effects of BDNF have been described in different preparations (Gottmann et al., 2009), this kind of enhancement appears predominantly of pre-synaptic origin, since basal glutamate release is stimulated by concomitant increases of the size of the readily releasable vesicle pool and the probability of vesicle release (Valente et al., 2012). Together, these results support the idea that Kidins220 is critically involved in the pre-synaptic BDNF signaling pathway acting on glutamate release (Figure 1A) as well as in post-synaptic TrkB-dependent retrograde signaling events acting on GABA release (Figure 1Ba).

**Figure 2:**
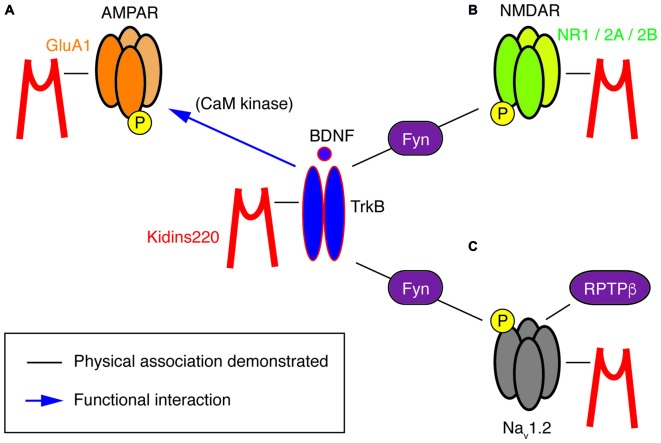
**A potential “TrkB/BDNF—Kidins220—ion channel” network.** This cartoon summarizes the known physical (black lines) and functional (blue arrows) interactions at present demonstrated for Kidins220, the NT receptor TrkB and its ion channel targets, i.e., subunits of AMPA-type and NMDA-type glutamate receptors as well as Na_v_ channels. **(A)** In the case of the Kidins220-AMPAR interaction, it is known that Kidins220 modulates the surface expression and phosphorylation state of the GluA1 subunit. Phosphorylation of the same subunit is known to be modulated by TrkB activation via CaM kinase. **(B)** Kidins220 interacts with the NR1, NR2A and NR2B subunits of NMDAR. TrkB activation modulates NMDAR phosphorylation via Fyn kinase. **(C)** Kidins220 interacts with Na_v_1.2 modulating channel kinetics and voltage-dependence. TrkB activation modulates Na_v_1.2 channel function via phosphorylation mediated by Fyn kinase, while dephosphorylation is mediated by receptor-type protein tyrosine phosphatase β (RPTPβ). For details refer to the main text.

In other studies, a direct relation to TrkB/BDNF signaling events is missing, yet a survey of the literature suggests hidden links that may deserve further investigation, in particular regarding the association of Kidins220 with subunits of two main classes of post-synaptic glutamate receptors. Starting from the observation that basal synaptic transmission was slightly increased in hippocampal slices prepared from 1-month-old *ARMS*^+/−^ mice, Arévalo et al. (2010) proposed that Kidins220 associates with the AMPA-type glutamate receptor subunit A1 (GluA1) and regulates its phosphorylation state and localization. Accordingly, Kidins220 overexpression or knock-down in rat organotypic brain slices caused inverse changes in GluA1 surface expression and in the amplitude of AMPA receptor-mediated EPSCs (Arévalo et al., 2010). Moreover, it is tempting to relate the Kidins220-GluA1 association also to long-term potentiation (LTP) of excitatory responses, since LTP at hippocampal Schaffer collateral—*Cornu Ammonis 1* (CA1) synapses was increased in 3–6-month-old *ARMS*^+/−^ mice (Wu et al., 2010). LTP at this synapse has been predominantly attributed to changes in the number and biophysical properties of AMPA receptors (Lee and Kirkwood, 2011). Notably, *ARMS*^+/−^ hippocampal slices and *Kidins220*-depleted neurons showed increased GluA1 phosphorylation at two serine residues, S831 and S845 (Arévalo et al., 2010), both of which are known to contribute to LTP induction at Schaffer collateral-CA1 synapses (Lee et al., 2003, 2010). These observations are circumstantial, as a direct link connecting the Kidins220-GluA1 association to LTP is still missing. Furthermore, increased LTP in *ARMS*^+/−^ mice was not due to reduced Kidins220 levels *per se*, but required further activity-dependent proteolytic degradation mediated by calpain protease (Wu et al., 2010), suggesting that the absolute value of the Kidins220 steady-state level may be important for the dynamic modulation of synaptic plasticity at glutamatergic synapses. Further work is needed to clarify the position of Kidins220 in these molecular pathways, also considering that Kidins220 associates both with TrkB and GluA1 receptors (Figure 2). BDNF is a potent regulator of hippocampal LTP with numerous roles (Leal et al., 2014). Recently, it has been demonstrated that BDNF regulates synaptic AMPA receptor incorporation in a form of spike-timing-dependent LTP at Schaffer collateral inputs (Edelmann et al., 2015). It has also been reported that short-term treatment with BDNF causes an increase in GluA1 surface expression in rat hippocampal neurons, mediated by the activation of the Ca^2+^/calmodulin-dependent (CaM) kinase pathway (Fortin et al., 2012) and phosphorylation of GluA1 at S831 (Caldeira et al., 2007; Figure 2). With our current knowledge, we can only take note of the unexpected fact that the effect of reduced Kidins220 levels appears to mirror the BDNF effect on GluA1 phosphorylation and surface expression (Figure 1Bb). It also remains to be determined which may be the consequences of the Kidins220-GluA1 association for the biophysical properties of the respective AMPA-type glutamate receptors.

Furthermore, Kidins220 was also shown to associate with the NR1, NR2A and NR2B subunits of neuronal N-methyl-D-aspartate (NMDA) receptors (López-Menéndez et al., 2009), a class of glutamate receptors particularly critical for LTP induction, due to their specific functional properties combining glutamate- and voltage-gated activation with calcium permeability. Unfortunately, possible roles of the Kidins220 association in NMDAR trafficking or function are at present completely unexplored. The situation is different for the TrkB/BDNF system, which may therefore guide future efforts related to the role of Kidins220 in this context. BDNF is known to stimulate the rapid phosphorylation of the NR1 and NR2B (but not NR2A) subunits (Suen et al., 1997; Lin et al., 1998) and increase the open probability of NMDARs, ultimately leading to an enhancement of excitatory synaptic transmission (Levine et al., 1998). Similar tyrosine phosphorylation of NR2B has also been observed after LTP induction in the rat dentate gyrus (Rostas et al., 1996). A subsequent study showed the importance of BDNF-stimulated NR2B phosphorylation for spatial memory formation and suggested the Src-family tyrosine kinase Fyn, which interacted with both TrkB and NR2B, as a possible link (Figure 2; Mizuno et al., 2003). However, a direct connection between BDNF-stimulated NMDAR phosphorylation and hippocampal LTP remains to be demonstrated.

In summary, the studies related to modified (but not constitutively abolished) *Kidins220* expression reveal a picture in which basal synaptic transmission in both GABAergic and glutamatergic synapses are affected in opposite ways: while post-synaptic responses in the former were reduced by *Kidins220* knockdown (Sutachan et al., 2010), responses in the latter were increased (Arévalo et al., 2010; Wu et al., 2010). Contrarily to what may be expected from these results, hippocampal neurons derived from full *Kidins220* knockout embryos did not show an impairment in basal synaptic transmission (Cesca et al., 2012; Scholz-Starke et al., 2012). It is conceivable that the lack of Kidins220 in these neurons may be compensated by homeostatic mechanisms to various extents, depending on its specific function in the process under study. Importantly, recordings on *Kidins220^−/−^* neurons revealed an entirely novel function of the Kidins220 protein in the control of synaptic plasticity, which apparently cannot be covered by compensatory mechanisms. It should be noted that this function (similarly to a further one related to neuronal excitability, which is discussed below) was associated with GABAergic hippocampal neurons, but apparently absent in glutamatergic neurons. Inhibitory post-synaptic currents (IPSCs) of *Kidins220^−/−^* neurons recovered considerably faster from synaptic depression than those recorded from wild-type neurons (Scholz-Starke et al., 2012). In response to two different stimulation paradigms, paired-pulse and long-lasting train stimulation, the kinetics of recovery of wild-type IPSCs was biphasic, displaying fast and slow components similar to what has been reported for IPSCs in collicular neurons and hippocampal basket cell—granule cell synapses (Kraushaar and Jonas, 2000; Kirischuk et al., 2002). Contrarily, the slow component of recovery for *Kidins220^−/−^* IPSCs was consistently reduced in both paradigms, while the fast component was unaffected. In wild-type neurons, the slow component was independent of synaptic vesicle depletion, but apparently linked to a transient reduction of vesicle release probability (Scholz-Starke et al., 2012). Therefore, these data suggest an essential role for Kidins220 in the transient, activity-dependent reduction of GABA release in hippocampal synapses (Figure 1A), but the exact mechanisms remain to be determined. Also in view of this novel function in synaptic plasticity, it may be interesting to transiently modify Kidins220 expression specifically in GABAergic neurons.

### Kidins220 as a Target of Neuronal Activity

In addition to its role as a player participating in the control of neuronal activity, it turned out that Kidins220 itself is a *target* of proteolytic degradation mediated by Ca^2+^-dependent calpain proteases, the activation of which is triggered by neuronal activity. Calpains do not degrade their targets completely, unlike typical cellular proteases, but function to regulate their activity by partial cleavage, thereby contributing to synaptic plasticity and neurotoxicity (Baudry et al., 2013). As a calpain target, Kidins220 is in the company of TrkB and numerous synaptic proteins, among which *SNA*P (Soluble NSF Attachment Protein) *re*ceptor (SNARE) proteins, glutamate receptor subunits, protein kinases, cytoskeletal and other scaffold proteins, just to name a few (Baudry et al., 2013). Kidins220 degradation was observed in response to excitotoxic overstimulation of cortical NMDARs, leading to neuronal death (López-Menéndez et al., 2009), but also following physiological activity in hippocampal neurons triggered by glutamate or KCl-elicited depolarization (Wu et al., 2010). Chronic activity stimulation by the GABA_A_ receptor antagonist bicuculline also caused a small decrease of Kidins220 protein levels in hippocampal neurons (Cortés et al., 2007). Although the mechanisms leading to Kidins220 downregulation are different in these studies, they all point to activity-dependent proteolytic Kidins220 degradation. This could be relevant in cases of pathological hyperexcitation, such as epileptic seizures, which cause, amongst many effects, also an increase in BDNF. Interestingly, calpain activity has been recently shown to affect the availability of other proteins controlling neuronal excitability, such as the K^+^/Cl^-^ co-transporter KCC2, during neonatal seizures (Puskarjov et al., 2015). Conversely, Kidins220 amounts tended to increase in neurons subjected to chronic activity blockade by tetrodotoxin, a potent inhibitor of voltage-gated Na^+^ (Na_v_) channels (Cortés et al., 2007). In this case, the underlying mechanism is at present unknown, yet Kidins220 accumulation may be simply due to reduced proteolytic degradation under conditions of activity blockade. Alternatively, it might be related to homeostatic synaptic scaling operating to restore normal synaptic activity under these conditions (Turrigiano, 2008). Once again, BDNF is among the factors regulating synaptic scaling. In fact, TrkB receptor inhibition mimics the effects of chronic activity blockade, and further, scaling up of synaptic strength is prevented by application of exogenous BDNF (Rutherford et al., 1998). In any case, it appears certain that Kidins220 is a *target* of activity-dependent regulation, while there is no indication for a possible role as activity *sensor*, as initially proposed by Cortés et al. (2007) on the basis of the reciprocal relationship between neuronal activity and Kidins220 levels and further data showing that *Kidins220* knock-down enhanced synaptic activity. While the mechanisms responsible for the enhancement were not further specified in this study, it seems now clear, in the light of subsequent studies discussed above, that they combined an increase of glutamatergic synaptic transmission (Wu et al., 2010) with a decrease of GABA_A_ receptor-mediated inhibition (Sutachan et al., 2010).

### A Novel Role of Kidins220 in the Control of Neuronal Excitability

Constitutive *Kidins220* ablation also affected the intrinsic excitability of GABAergic hippocampal neurons. Specific alterations in action potential shape strongly suggested an increased sodium conductance in *Kidins220^−/−^* inhibitory neurons, possibly caused by aberrant activity of Na_v_ channels (Cesca et al., 2015). Biochemical studies confirmed that Kidins220 associates with alpha subunits of native Na_v_ channels in the brain and specifically with sodium channels formed by the major brain Na_v_ channel alpha subunit Na_v_1.2 in human embryonic kidney (HEK)293 cells. Recordings of sodium currents mediated by heterologously expressed Na_v_1.2 further revealed dramatically slowed channel kinetics and shifted voltage-dependence in Kidins220-coexpressing cells, indicating that Kidins220 association can have unexpectedly strong effects on both Na_v_ channel activation and fast inactivation processes (Cesca et al., 2015). These data suggest that Kidins220 exerts a negative influence on Na_v_ channel activity in GABAergic neurons (Figure 1A).

This kind of modulation differed in several aspects from the regulation of Na_v_1.2 channels by Fyn tyrosine kinase in response to the activation of the TrkB/ BDNF pathway (Ahn et al., 2007). First, the effects depended solely on Kidins220 co-expression, but not on further constituents of the TrkB signaling pathway or BDNF application. Second, Na_v_1.2 phosphorylation by Fyn did not affect channel activation, but only fast inactivation, and third, it accelerated inactivation and shifted its voltage-dependence towards negative membrane potentials, i.e., in the opposite direction compared to Kidins220. The activity of brain Na_v_1.2 channels appears to be modulated by Fyn-mediated phosphorylation, which can be reversed by dephosphorylation catalyzed by the receptor-type protein tyrosine phosphatase β (RPTPβ; Figure 2; Ratcliffe et al., 2000). A radically different mode of BDNF action has been proposed for the alpha subunit Na_v_1.9, in which TrkB activation directly elicits the rapid activation of sodium currents by an as yet unknown mechanism (Blum et al., 2002). Although these results have not been reproduced by other groups and are therefore not generally accepted, it is notable that focal BDNF application elicited fast calcium transients in the dendrites of hippocampal neurons, which required the activity of Na_v_ channels, in addition to TrkB receptors and voltage-dependent Ca^2+^ channels (Lang et al., 2007). Future studies related to cell type/subunit specificities and the molecular mechanism of the Kidins220-Na_v_ channel interaction may also reveal if and how it relates to the Fyn-mediated modulation and more generally to the TrkB/BDNF pathway. A further aspect of the interaction concerns its sub-cellular localization within the neuron. Na_v_ channel clustering at the axon initial segment and nodes of Ranvier is crucial for reliable action potential generation and conduction. Clustering is achieved by the adaptor protein ankyrin-G, which links Na_v_ channels to the actin/spectrin cytoskeleton (Zhang and Bennett, 1998; Garrido et al., 2003). Similarly, the ankyrin repeats present in the Kidins220 N-terminus may be involved in Na_v_ channel association and possibly interfere with normal channel clustering.

At the single-neuron level, *Kidins220^−/−^* GABAergic neurons displayed increased excitability, which manifested itself as a reduction of threshold currents required to elicit action potentials and increased firing frequencies compared to wild-type neurons (Cesca et al., 2015). Misregulation of Na_v_ channels contributes to some extent to these phenotypic changes, but given the complexity of neuronal firing, one cannot exclude that further, as yet unidentified molecular mechanisms will add to it. Finally, multi-electrode array recordings of *Kidins220^−/−^* hippocampal networks revealed reduced spiking activity in response to low-frequency pulse stimulation (Cesca et al., 2015), suggesting that the phenotypic changes observed in *Kidins220^−/−^* GABAergic neurons translate into specific changes of network excitability. These results were consistent with the idea that reverberating network excitation was suppressed by a potentiation of inhibitory neuronal circuits. It remains to be determined if the occurrence of two gain-of-function phenotypes specifically in GABAergic *Kidins220^−/−^* neurons identifies a regulatory role of the protein in the weight of synaptic inhibition and ultimately in the balance between excitation and inhibition in neuronal networks.

## Kidins220 Functions Related to Pathologies

Studies performed on Kidins220 mutant mice indicate that the alteration of Kidins220 protein levels impairs neuronal survival and development. The complete ablation of Kidins220 led to embryonic death, which was associated to extensive apoptosis in the CNS and PNS and to cardiovascular abnormalities (Cesca et al., 2011, 2012). A partial reduction of Kidins220 levels was sufficient to cause defects in spine turnover and synaptic plasticity, with significant repercussions on higher functions such as learning and memory, when studied in adult mice (Wu et al., 2009, 2010; Arévalo et al., 2010; Duffy et al., 2011). Taken together, the results of these studies strongly support the idea that alterations of the *KIDINS220* gene and/or the Kidins220 protein may associate with human (neuro)pathologies. Although the literature concerning this aspect of Kidins220 function is still limited, several studies have started to investigate the genetic and molecular pathways linking Kidins220 to the onset of various diseases. For example, Kidins220 is overexpressed in human samples of melanoma (Liao et al., 2007, 2011), a tumor of neural crest origin, and of neuroblastoma (Rogers and Schor, 2013a,b; Jung et al., 2014), a cancer type affecting the PNS. In both cases, *Kidins220* behaves as an oncogene, affecting the ability of cancer cells to survive, proliferate and migrate/metastasize. Increased Kidins220 levels have also been observed in human Alzheimer’s Disease (AD) samples (López-Menéndez et al., 2013). Here, Kidins220 accumulated with hyperphosphorylated Tau protein, probably contributing to the defective NT signaling observed in this pathology. Alterations of *KIDINS220* gene expression have been found in several genetic screens in humans. For example, a microarray-based expression profiling of dopaminergic neurons isolated from the substantia nigra of Parkinson’s Disease (PD) patients revealed that Kidins220 levels were significantly decreased compared to controls (Simunovic et al., 2009). Another study analyzed the blood transcriptome from Autism Spectrum Disorder (ASD) patients, and found increased levels of Kidins220 mRNA in ASD patients. Interesting, genes belonging to the NT pathways were overall the most represented amongst the affected genes (Kong et al., 2012). Finally, a very recent study was conducted on a small number of schizophrenia patients, by means of high-coverage targeted exome capture on a small number of NT-related genes from blood leukocytes. Interestingly, two *KIDINS220* missense polymorphisms and one novel gene variant were identified in 5 out of 48 schizophrenia patients (Kranz et al., 2015). One of these polymorphisms has been subsequently associated to reduced prefrontal rostralization in schizophrenia patients, compared to healthy control subjects (Malaspina et al., 2016). A schematic summary of the pathologies that have been associated with Kidins220 to date is reported in Table 1.

**Table 1:** **Pathologies associated to mutations of the *Kidins220* gene or alterations of Kidins220 protein levels**.

Disease	Mutation	Physiological effects	Organism	Reference
Melanoma	Increased levels	Cell survival, anchorage-independent growth/metastasis	Human, mouse	Liao et al. (2007); Liao et al. (2011)
Neuroblastoma	Increased levels	N-type to S-type transition, NGF-mediated signaling, cell proliferation	Human, mouse	Rogers and Schor (2013a,b), Jung et al. (2014)
Pediatric high-grade glioma	Intragenic copy number breakpoint	n.d.	Human	Carvalho et al. (2014)
Alzheimer’s disease	Increased levels	Decreased Kidins220 clearance and impaired NT signaling	Human	López-Menéndez et al. (2013)
Parkinson’s disease	Reduced levels	n.d.	Human	Simunovic et al. (2009)
Autism spectrum disorders	Copy number variation (increase)	n.d.	Human	Kong et al. (2012)
Autism spectrum disorders	Deletion of genomic region	n.d.	Human	Pinto et al. (2014)^a^
Schizophrenia	Two missense polymorphisms (A1299G, A557V), one novel variant (H1085R)	n.d.	Human	Kranz et al. (2015) and Malaspina et al. (2016)

*n.d., not determined. ^a^Deletion of the genomic region containing Kidins220 was identified in 3 out of 2446 cases analyzed, however it did not pass subsequent quality controls and was not further investigated*.

## Outlook

The scaffold protein Kidins220, originally described as the first physiological substrate of Protein Kinase D (PKD) and as an immediate down-stream target of NT receptor kinases, has emerged as a novel player in the control of neuronal activity. However, our understanding of this role is currently far from being complete, and it is also unclear how it relates to the recent identification of Kidins220 in several neurological diseases. This summary may serve to focus on gaps in our knowledge on the subject and possibly to guide future investigations.

At the cellular level, it will be important to clarify which cases of recognized Kidins220-mediated regulation are *de facto* part of TrkB/BDNF signaling events and which are not. There is a multitude of mechanisms by which BDNF impacts on synaptic transmission and membrane excitability and which might also involve Kidins220. In fact, the list of ion channels targeted by the BDNF signaling pathway has grown considerably in recent years and now includes, apart from the above-mentioned NMDARs (Levine et al., 1998) and voltage-gated sodium channels (Ahn et al., 2007), also TRP-type non-selective cation channels (Li et al., 1999; Amaral et al., 2007), several types of potassium channels (Rogalski et al., 2000; Tucker and Fadool, 2002; Nieto-Gonzalez and Jensen, 2013) and voltage-gated calcium channels (Baydyuk et al., 2015). The current picture that has emerged from the available data shows that Kidins220 associates with subunits of voltage-gated sodium channels and two classes of neuronal glutamate receptors, which are, for their part, strongly modulated by the TrkB/BDNF system (Figure 2). This raises the question whether the target proteins are part of the same local signaling complex or, alternatively, if there are distinct, spatially separated Kidins220 pools operating with TrkB and different types of membrane receptors. In this context, it may also be relevant to note that Kidins220 has been found associated to lipid rafts (Cabrera-Poch et al., 2004), for which a connection to NT signaling in synapses has been proposed (Zonta and Minichiello, 2013). More work is needed to investigate a possible Kidins220 association with other ion channels known to be modulated by BDNF. It is also unknown whether a connection exists between Kidins220 and Fyn kinase, which has been identified as a mediator in the BDNF modulation of Na_v_1.2 and NMDARs (Mizuno et al., 2003; Ahn et al., 2007).

Among the plethora of known Kidins220 interactions, for some it is known that Kidins220 can induce post-translational modifications of partner proteins, even though this aspect has been investigated only in a minority of studies (Table 2). Since Kidins220 does not seem to possess any kind of enzymatic activity, such effects must necessarily be indirect, most probably through the assembly of multi-protein complexes where the modifying enzyme and its target protein are brought in close proximity by means of the Kidins220 scaffold. This is indeed a topic worth pursuing, since it may give a strong contribution to our understanding of the mechanisms by which Kidins220 regulates various aspects of synaptic plasticity. A further layer of complexity is given by the recent identification of a number of different Kidins220 isoforms, which show age- and tissue-specific distribution (Schmieg et al., 2015). Such variants determine the intracellular localization of the Kidins220 protein itself and of its molecular partners, as shown for the TrkA receptor (Schmieg et al., 2015). However, this field of investigation is relatively new and many of the already identified interactions may turn out to be isoform-specific.

**Table 2:** **Kidins220 interacting partners, and post-translational modifications (PTMs) triggered by the interactions**.

Interacting partner	Binding site on Kidins220	Binding site on interacting partner	Reciprocal PTMs	Reference
AMPAR-GluA1	Transmembrane domains	Not the C-terminus	Kidins220 negatively regulates GluA1 phosphorylation at Ser831 and Ser845	Arévalo et al. (2010)
**α**- and **β**2-syntrophin	PDZ-binding motif	PDZ domain	n.d.	Luo et al. (2005)
B cell antige *n* receptor (BCR)	n.d.	n.d.	n.d.	Fiala et al. (2015)
B-Raf	n.d.	n.d.	n.d.	Deswal et al. (2013)
Caveolin-1	n.d.	n.d.	n.d.	Jean-Mairet et al. (2011)
CrkL	Proline-rich domain (residues 1089–1093)	SH3 domain (constitutive binding); SH2 domain (by binding phospho- Tyr^1096^)	n.d.	Arévalo et al. (2004, 2006)
EphA4	n.d.	n.d.	Kidins220 and α-syntrophin induce EphA4 Tyr phosphorylation; EphA4 induces Kidins220 Tyr phosphorylation	Luo et al. (2005)
ICAM-3	n.d.	n.d.	n.d.	Jean-Mairet et al. (2011)
IKKα/β	n.d.	n.d.	n.d.	Singh et al. (2015)
Kinesin 1	KIM motif	KLC residues 83–296	n.d.	Bracale et al. (2007)
MAP1a, MAP1b, MAP2	Residues 760–1762	MAP1a LC2, MAP1b LC1	Kidins220 induces phosphorylation of MAP1b HC, as well as an increase in its total levels	Higuero et al. (2010)
Na^+^ channels, Voltage-gated	n.d.	n.d.	n.d.	Cesca et al. (2015)
NMDA receptor subunits NR2A, NR2B, NR1	n.d.	n.d.	NMDAR overactivation reduces Kidins220 levels	López-Menéndez et al. (2009)
Olfactomedin 1 (Olfm1)	n.d.	n.d.	n.d.	Nakaya et al. (2013)
p75^NTR^	Residues 1512–1762	Juxtamembrane region (residues 300–315)	n.d.	Kong et al. (2001) and Chang et al. (2004)
PDZ-GEF1	Indirect binding through S-SCAM	n.d.	n.d.	Hisata et al. (2007)
Pdzrn3	PDZ-binding motif	First PDZ domain (residues 249–339)	n.d.	Andreazzoli et al. (2012)
Protein Kinase D (PKD)	n.d.	n.d.	PKD phosphorylates Kidins220 on Ser^919^ upon phorbol ester treatment	Iglesias et al. (2000)
Septin 5	Residues 1603–1715	N-terminal region (residues 125–213)	n.d.	Park et al. (2010)
Sortin nexin 27 (SNX27)	PDZ-binding motif	PDZ domain	n.d.	Steinberg et al. (2013)
Statmins (SCG10, SCLIP)	Ankyrin repeats	n.d.	Kidins220 induces Ser phosphorylation of statmins	Higuero et al. (2010)
S-SCAM	PDZ-binding motif	PDZ4 domain	n.d.	Hisata et al. (2007)
T-cell receptor (TCR)	n.d.	n.d.	n.d.	Deswal et al. (2013)
Trio	Ankyrin repeats	N-terminus (spectrin repeats)	n.d.	Neubrand et al. (2010)
TrkA, TrkB, TrkC	Transmembrane domain	Transmembrane domains	n.d.	Kong et al. (2001) and Arévalo et al. (2004)
Tubulin-βIII, acetylated and tyrosinated α-tubulin	n.d.	n.d.	n.d.	Higuero et al. (2010)
VEGFR2, VEGFR3	n.d.	n.d.	n.d.	Cesca et al. (2012)

*n.d., not determined*.

Some Kidins220 effects on synaptic plasticity and membrane excitability were observed specifically in GABAergic, but not glutamatergic neurons (Scholz-Starke et al., 2012; Cesca et al., 2015). Since Kidins220 is expressed in both excitatory and inhibitory neurons, this specificity may be related to the cell-specific expression of Kidins220-interacting proteins or, alternatively to the differential expression of *KIDINS220* splice variants (Schmieg et al., 2015). It is important to underline that the effects of these newly identified splice variants, as well as of specific disease-related *KIDINS220* mutations, on the cellular localization and function of the protein are at present completely unexplored. Finally, future studies on the role of Kidins220 in the control of neuronal excitability will also have to consider the complex interaction between neurons and glial cells within the nervous system, also in view of the important role that astrocytes (Bergami et al., 2008), oligodendrocytes (Wong et al., 2013) and microglial cells (Parkhurst et al., 2013) play in the modulation of numerous aspects of BDNF physiology in the CNS and PNS.

At the level of the whole organism, it appears clear that a dysregulation of Kidins220 physiology, may it be caused by a variation of protein levels or by amino acid mutations, is cytotoxic and potentially pathogenic. Interestingly, the presynaptic roles of Kidins220 (Figure 1A) have been identified in studies conducted on Kidins220^−/−^ neurons, while its function at the post-synaptic level (Figure 1B) has been characterized mostly by acute and transient manipulation of Kidins220 levels. Thus, although it is likely that Kidins220 exerts all the above-described functions under physiological conditions, it is tempting to speculate that the presynaptic effects observed in the absence of the protein are indicative of what may happen under pathological conditions, when Kidins220 protein levels are drastically reduced or absent because of loss-of-function mutations or genetic aberrations, while postsynaptic alterations may be the consequence of physiological, activity-dependent variations of Kidins220 levels. Although very little information is available so far concerning the molecular pathways involved, it is reasonable to speculate that some of the pathogenic effects may be due to aberrant NT signaling. However, possible effects on neuronal morphology, synaptic plasticity and membrane excitability should not be overlooked, especially in view of the data obtained from adult mice expressing reduced levels of this protein (see above; Wu et al., 2009, 2010; Arévalo et al., 2010; Duffy et al., 2011). In this respect, studies on conditional knockout mouse lines lacking Kidins220 in a tissue-specific fashion will be instrumental to unveil new roles of this protein in the onset and progression of a number of pathologies, inside and outside the nervous system. This is well exemplified by a very recent study, in which Kidins220 was specifically deleted in B cells (Fiala et al., 2015). Here, B cell receptor-mediated B cell activation was reduced, thus placing Kidins220 in a central position to modulate the immune response. Furthermore, it will be crucial to expand our knowledge of Kidins220 mutations by performing wider screenings, including larger populations of human patients and/or a wider range of pathologies.

## Author Contributions

JS-S and FC conceived and wrote the manuscript. FC prepared the figures. JS-S and FC approved the final version.

## Funding

This work was supported by a grant from the Compagnia di San Paolo (grant #2013.1014 to FC).

## Conflict of Interest Statement

The authors declare that the research was conducted in the absence of any commercial or financial relationships that could be construed as a potential conflict of interest.

## Abbreviations

AMPA: α-amino-3-hydroxy-5-methyl-4-isoxazolepropionic acid
BDNF: brain-derived neurotrophic factor
EPSC: excitatory post-synaptic current
GABA: gamma-aminobutyric acid
GluA1: glutamate receptor A1
IPSC: inhibitory post-synaptic current
Kidins220/ARMS: Kinase D interacting substrate of 220 kDa/ankyrin-repeat-rich membrane spanning
LTP: long-term potentiation
Na_v_: voltage-gated Na^+^ channel
NGF: nerve growth factor
NMDA: N-methyl-D-aspartate
NMDAR: NMDA receptor
NT: neurotrophin
Trk: tropomyosin-related kinase.
